# Immunometabolite L-2-HG promotes epigenetic modification of exhausted T cells and improves antitumor immunity

**DOI:** 10.1172/jci.insight.174600

**Published:** 2025-03-04

**Authors:** Yanying Yang, Xiaoyan Li, Fangming Liu, Mingyue Ma, Ying Yang, Chengchao Ruan, Yan Lu, Xiaoyang Li, Xiangdong Wang, Yinghong Shi, Zheng Zhang, Hua Wang, Zhouli Cheng, Duojiao Wu

**Affiliations:** 1 Department of Endocrinology, Zhongshan Hospital, and; 2 Department of Physiology and Pathophysiology, Shanghai Key Laboratory of Bioactive Small Molecules, State Key Laboratory of Medical Neurobiology, School of Basic Medical Sciences, Fudan University, Shanghai, China.; 3 Department of Endocrinology, Zhongnan Hospital of Wuhan University, Wuhan, China.; 4 Department of Oncology, the First Affiliated Hospital of Anhui Medical University, Hefei, China.; 5 Department of Geriatrics, Xinhua Hospital, Shanghai Jiao Tong University School of Medicine, Shanghai, China.; 6 Shanghai Key Laboratory of Lung Inflammation and Injury, Zhongshan Hospital, Fudan University, Shanghai, China.; 7 Institute of Metabolism and Regenerative Medicine, Digestive Endoscopic Center, Shanghai Sixth People’s Hospital Affiliated to Shanghai Jiao Tong University School of Medicine, Shanghai, China.; 8 Jinshan Hospital Center for Tumor Diagnosis & Therapy, Jinshan Hospital, Fudan University, Shanghai, China.; 9 Shanghai Institute of Hematology, State Key Laboratory of Medical Genomics, National Research Center for Translational Medicine at Shanghai, Ruijin Hospital Affiliated to Shanghai Jiao Tong University School of Medicine, Shanghai, China.; 10 Liver Surgery Department of Zhongshan Hospital, Fudan University, Shanghai, China.; 11 Institute for Hepatology, National Clinical Research Center for Infectious Disease, Shenzhen Third People’s Hospital, The Second Affiliated Hospital, School of Medicine, Southern University of Science and Technology, Shenzhen, China.; 12 Shanghai Institute of Infectious Disease and Biosecurity, Fudan University, Shanghai, China.

**Keywords:** Immunology, Metabolism, Cancer immunotherapy, T cells

## Abstract

This study aimed to explore the potential correlation between the metabolic intermediate L-2-hydroxyglutarate (L-2-HG) and T cell exhaustion, as well as the underlying mechanisms involved. In this study, we investigated the presence of exhausted T (Tex) cells in patients under certain conditions: HIV infection, chronic leukemia, and hepatocellular carcinoma. To gain insights into the epigenetic signatures and transcriptome changes in Tex cells, we employed a combination of RNA-seq and ATAC-seq analyses. To evaluate the impact of L-2-HG on mitochondrial function, differentiation, and antitumor capacity of Tex cells, we utilized in vitro cell culture experiments and animal tumor models. We observed mitochondrial depolarization and metabolic dysfunction in Tex cells, accompanied by a significant reduction in L-2-HG levels. Moreover, altered epigenetic characteristics were observed in Tex cells, including a substantial increase in H3K27me3 abundance. Culturing Tex cells with L-2-HG demonstrated improved mitochondrial metabolism, reduced H3K27me3 abundance, and enhanced memory T cell differentiation. In a mouse melanoma tumor model, L-2-HG–treated CD8^+^ T cells for adoptive therapy led to significantly reduced tumor volume and significantly enhanced effector function of T cells. The study revealed that L-2-HG acted as an immune metabolite through epigenetic modifications of Tex cells.

## Abstract 2

The study revealed L-2-HG acted as an immune metabolite through epigenetic modifications, reversed the progression of cell exhaustion, and improved the anti-tumor immunity of Tex.

## Graphical abstract

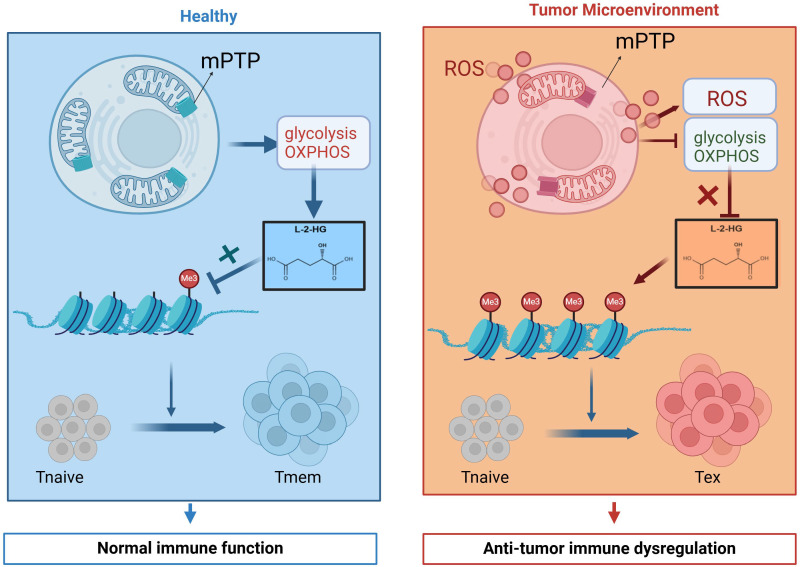

## Introduction

T cell exhaustion is a state of T cell dysfunction commonly observed in chronic viral infections and cancer, posing a significant obstacle to effective immune responses (1, 2). Clinical trials employing checkpoint blockade therapy to reverse T cell exhaustion have shown remarkable successes in cancer treatment, underscoring the importance of understanding the mechanisms that regulate exhausted T (Tex) cells in the context of cancer immunotherapy (3, 4). Specifically, Tex cells in cancer and chronic viral infections exhibit distinct gene expression patterns, characterized by impaired production of effector cytokines such as interferon γ (IFN-γ) and tumor necrosis factor (TNF), as well as increased expression of inhibitory receptors including programmed cell death protein 1 (PD1) and lymphocyte-activation protein 3 (LAG3), which are hallmark features of T cell exhaustion (5–7). Interestingly, CD8^+^ tumor-infiltrating lymphocytes (TILs) undergo progressive changes in chromatin architecture during the development of T cell exhaustion, as demonstrated by recent studies (8–10). Moreover, studies in both human and mouse models of chronic viral infection have revealed that CD8^+^ Tex cells acquire a state-specific epigenetic landscape (11). Furthermore, metabolic insufficiency, characterized by diminished glucose uptake and oxidative phosphorylation (OXPHOS), is a common characteristic of Tex cells in both chronic viral infections and tumors (12, 13). Additionally, the loss of mitochondrial activity and biogenesis is observed in Tex cells, which was attributed to reduced expression of proliferator-activated receptor γ coactivator 1α (PGC1α), a critical transcriptional coactivator responsible for supporting mitochondrial biogenesis and antioxidant responses (14). Therefore, the identification of targets to restore metabolic function and chromatin architecture in Tex cells is a crucial objective in the development of effective cancer immunotherapies.

Growing evidence suggests that metabolites accumulated in the tumor environment may play a causal role in tumor initiation and progression (15). These oncometabolites often accumulate due to mutations in genes encoding enzymes of the citric acid cycle, which are frequently recognized as driver mutations in tumorigenesis (15, 16). Gain-of-function mutations in isocitrate dehydrogenase 1 (IDH1) and IDH2 genes induce the neomorphic enzymatic production of 2-hydroxyglutarate (2-HG) (17, 18). Notably, 2-HG is a chiral molecule that exists in 2 enantiomeric conformations: D-(*R*) and L-(*S*) enantiomers. While IDH1/2 mutants exclusively produce D-2-HG, biochemical evidence suggests that L-2-HG can potently inhibit several α-ketoglutarate–dependent (α-KG–dependent) enzymes, including epigenetic modifiers (19, 20). D-2-HG has been shown to perturb transcriptional activity in the nuclear factor of activated T cells, leading to changes in the chromatin signature of CD8^+^ T cells, suppression of T cell cytotoxicity, and impaired IFN-γ signaling (21, 22). In contrast, L-2-HG accumulates in mouse CD8^+^ T cells in response to T cell receptor (TCR) triggering and hypoxia through a hypoxia-inducible factor 1α–dependent (HIF1α-dependent) mechanism (23, 24). The divergent effects of L-2-HG and D-2-HG on T cell function may be attributed to their differential impacts on glycolysis and varying inhibition constant (*K*_i_) values on target proteins such as lactose dehydrogenase (LDH) and α-KG–dependent enzymes (21, 25).

Our study unveiled that Tex cells within tumors exhibit impaired mitochondrial function and decreased levels of L-2-HG, a metabolite that influences epigenetic modification and gene expression. Additionally, we discovered differences in chromatin accessibility between PD1^+^CD8^+^ T cells and PD1^–^CD8^+^ T cells. Our findings demonstrated that reducing L-2-HG levels in T cells alters the H3K27me3 modification and expression of thymocyte selection–associated HMG box (*TOX*), ultimately determining the fate commitment of Tex cells. Conversely, replenishing L-2-HG levels could enhance mitochondrial fitness and augment the antitumor function of T cells by reducing *TOX* expression.

## Results

### Mitochondrial depolarization in Tex cells from patients with HCC.

**Figure 1:**
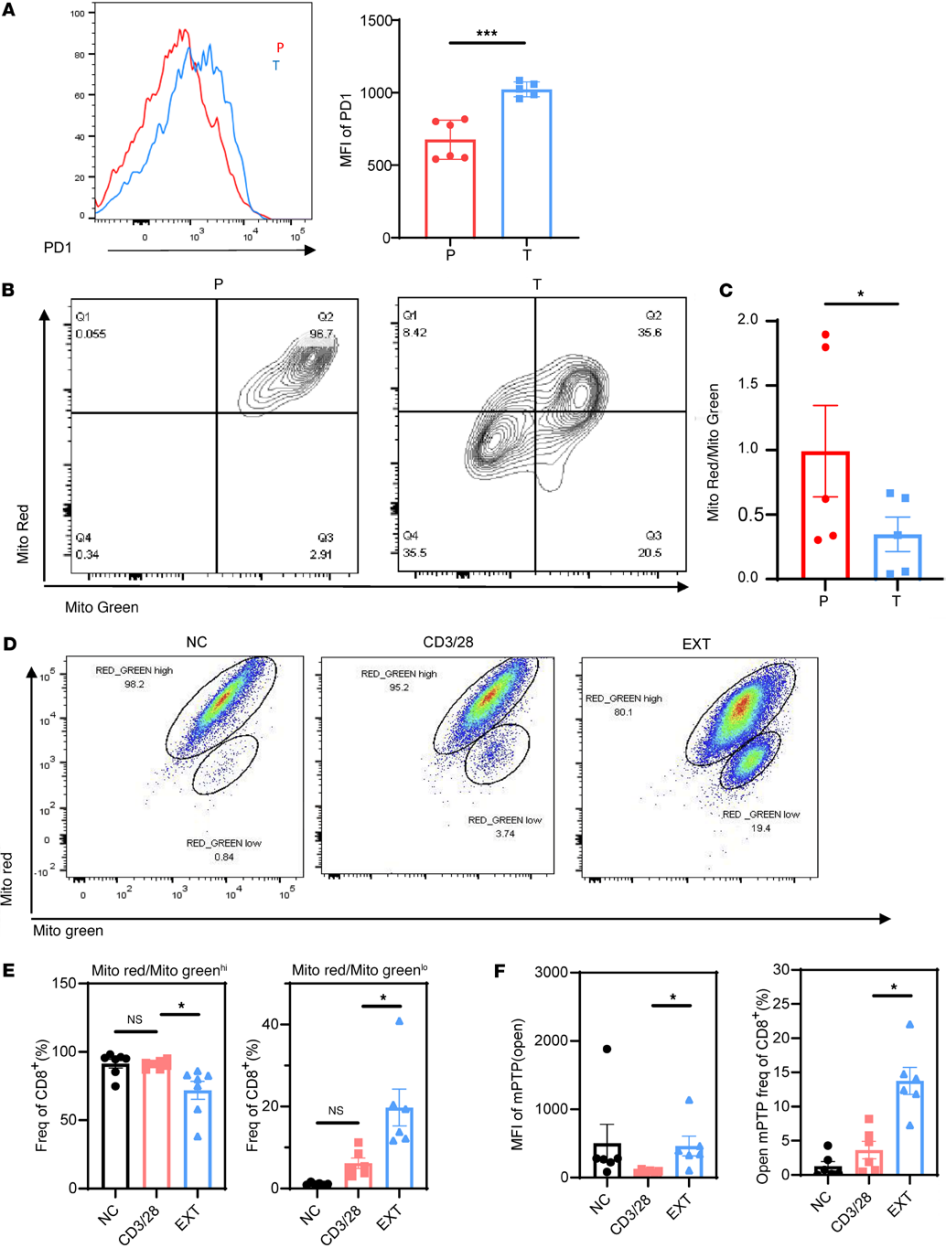
Mitochondrial membrane potential of T cells in tumor tissue and adjacent tissue of patients with HCC. (**A**) Flow cytometric representation of PD1 expressed by TILs isolated from paracancerous tissue (left) and tumor tissue (right) of patients with HCC. (**B**) Flow cytometric representation of mitochondrial mass and membrane potential of TILs isolated from paracancerous tissue (left) and tumor tissue (right) of patients with HCC detected by MitoGreen and MitoRed. (**C**) Histogram showing the MitoRed/MitoGreen ratio. (**D**) Flow cytometric representation of mitochondrial mass and membrane potential of T cells in the naive T cell negative control (NC) group (left), CD3/CD28-activated group (middle), and exhaustion group (right) detected by MitoRed and MitoGreen. (**E**) Proportion of MitoRed/MitoGreen^hi^ (left) and MitoRed/MitoGreen^lo^ (right) among total CD8^+^ T cells. (**F**) An mPTP detection kit was used to detect the MFI of open mPTP and proportion of open mPTP among total CD8^+^ T cells by T cells in the NC group, CD3/CD28-activated group, and exhaustion group; *n* ≥ 5 per group (**A**–**F**) and each point represents 1 sample. Data are represented as mean ± SEM. *P < 0.05; ***P < 0.001 by 2-tailed Student’s *t* test (**A** and **C**) or 1-way ANOVA (**E** and **F**). P, paracancerous tissue; T, tumor tissue; NC, T naive negative control group; CD3/28, CD3/CD28-activated group; EXT, exhaustion group; MFI, mean fluorescence intensity; MitoGreen: MitoTracker Green; MitoRed: MitoTracker Red; mPTP, mitochondrial permeability transition pore.

We found that tumor-infiltrating T cells in patients with hepatocellular carcinoma (HCC) exhibited a significant increase in PD1 protein expression, indicating T cell exhaustion (Figure 1A). Additionally, the mitochondrial characteristics of tumor-infiltrating T cells were altered. The assessment of mitochondrial membrane potential and mitochondrial mass in both para-cancerous tissue and tumor tissue demonstrated decreased mitochondrial activity in the tumor tissue. This was indicated by a reduced ratio of MitoRed to MitoGreen fluorescence in tumor-infiltrating T cells (Figure 1, B and C). These findings suggest that the mitochondria of tumor-infiltrated T cells experienced depolarization and dysfunction. This observation was supported by the data in Figure 1, B and C, and Supplemental Figure 1A; supplemental material available online with this article; https://doi.org/10.1172/jci.insight.174600DS1

To gain further insights into the mitochondrial status of Tex cells, an in vitro T cell exhaustion model was established (26) (Supplemental Figure 1B). In this model, the mRNA levels of co-suppressive molecules *CTLA4*, *TIM3*, and *PDCD1* were significantly elevated in Tex cells compared with the control group (Supplemental Figure 1C). The Tex cells also exhibited impaired effector function, as indicated by reduced mRNA and protein levels of effector molecules IFN-γ, TNF-α, perforin, and granzyme B (GZMB) (Supplemental Figure 2, A–E). These findings confirmed the successful establishment of the exhaustion model.

The mitochondrial status in the exhaustion model was further investigated by examining the levels of MitoRed and MitoGreen fluorescence. Tex cells showed lower levels of MitoRed and MitoGreen fluorescence compared with the CD3/CD28-activated group (Supplemental Figure 2F). Flow cytometric analysis revealed 2 distinct cell populations based on their MitoRed/MitoGreen ratio: MR/MG^hi^ (higher ratio) and MR/MG^lo^ (lower ratio) populations (Figure 1D). Importantly, the exhausted group had a significant decrease in the number of MR/MG^hi^ cells and a significant increase in the number of MR/MG^lo^ cells compared with the CD3/CD28-activated group, consistent with the observations in HCC samples (Figure 1, D and E). The MR/MG^hi^ population represented T cells with better mitochondrial polarization and higher membrane potential per unit mitochondrial mass, while the MR/MG^lo^ population represented T cells with mitochondrial depolarization and lower membrane potential per unit mitochondrial mass (27).

Additionally, we observed an abnormal accumulation of reactive oxygen species (ROS) and a decrease in tetramethylrhodamine ethyl ester (TMRM) levels in Tex cells, suggesting mitochondrial depolarization in Tex cells (Supplemental Figure 2G). The mitochondrial permeability transition pore (mPTP) is a channel formed by components of the inner and outer mitochondrial membranes (28). It plays a role in regulating the uptake and release of calcium ions by mitochondria and switches between on and off states. mPTP is associated with the release of mitochondrial components during cell death and affects mitochondrial permeability (29). Our investigation revealed an increase in mPTP opening in Tex cells (Figure 1F).

### The concentration of L-2-HG decreased significantly in Tex cells.

The abnormal opening of mPTP and the elevated production of ROS can result in the release of cytochrome *c* from mitochondria and the loss of mitochondrial membrane potential. Based on this, we propose that the abnormal mPTP opening in Tex cells, potentially influenced by increased ROS, further contributes to mitochondrial depolarization. The state of mitochondrial polarization serves as an indicator to evaluate mitochondrial dysfunction. Taken together, mitochondrial depolarization, impaired membrane potential, and ROS production are distinct characteristics of Tex cells in chronic viral infections and tumor microenvironments, significantly impacting their metabolic function and activity.

**Figure 2:**
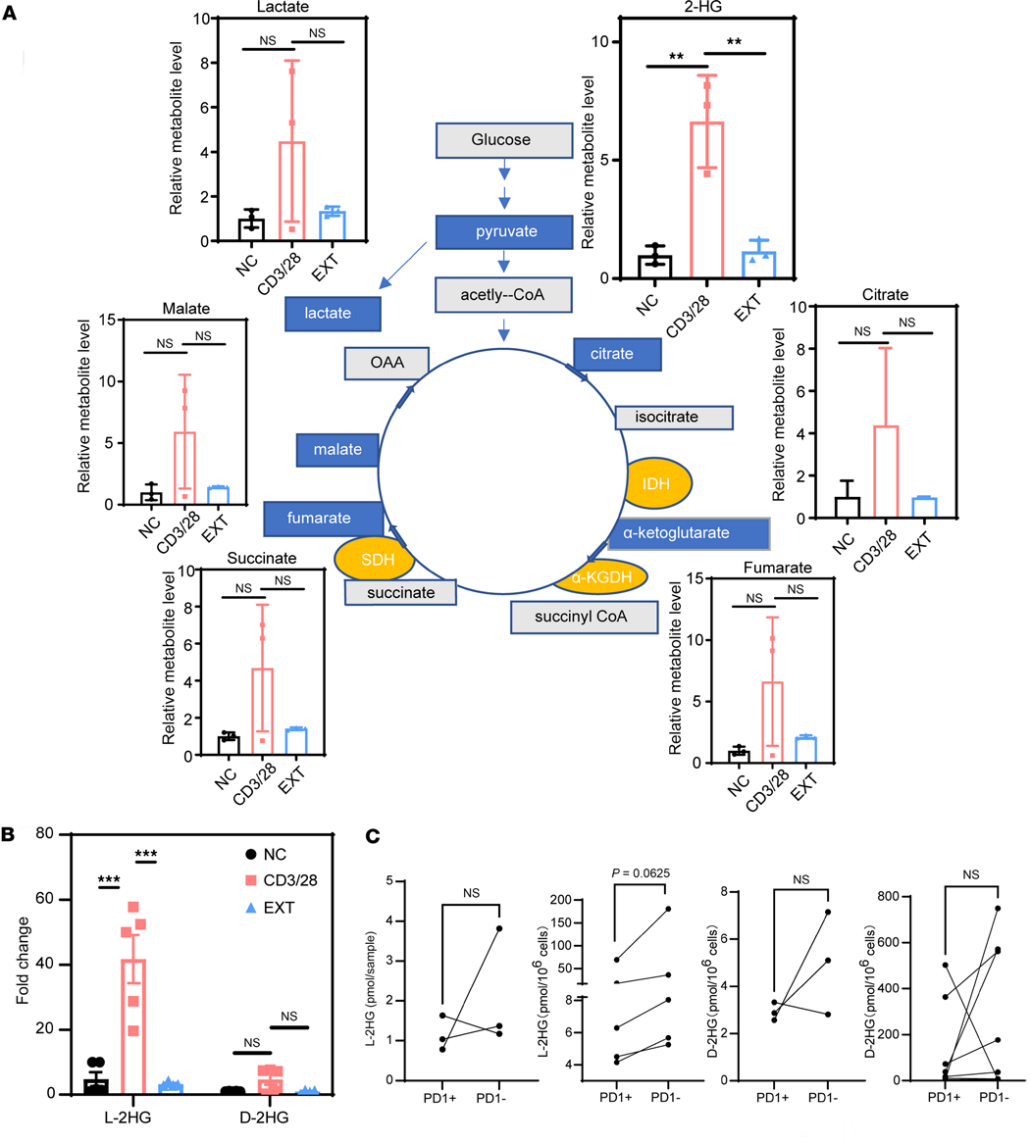
Metabolic imbalance of Tex cells. (**A**) Detection of Krebs cycle metabolites in NC, CD3/CD28-activated, and exhaustion group by GC-MS. (**B**) Detection of 2-HG levels by GC-MS in NC, CD3/CD28-activated, and exhaustion group. (**C**) L-2-HG and D-2-HG were detected by UHPLC-MS/MS in PD1^+^CD8^+^ and PD1^–^CD8^+^ T cells isolated from PBMCs of HIV patients (left) and leukemia patients (right). *n* ≥ 3 per group (**A**–**C**) and each point represents 1 sample. Data are represented as mean ± SEM. **P < 0.01; ***P < 0.001 by 1-way ANOVA (**A** and **B**) or 2-tailed Student’s *t* test (**C**). OOA, oxaloacetate; SHD, succinate dehydrogenase; α-KGDH, α-ketoglutarate dehydrogenase complex; IDH, isocitrate dehydrogenase [NADP(+)]; L-2-HG, L-2-hydroxyglutarate; D-2-HG, D-2-hydroxyglutarate.

In order to investigate this further, we conducted analysis by ultra-high-performance liquid chromatography coupled with triple quadrupole mass spectrometry (UHPLC-MS/MS) to examine the levels of key metabolites related to the Krebs cycle and amino acids in effector T (Teff) and Tex cells (Figure 2A and Supplemental Figure 3A). Our findings revealed a significant increase in 2-HG levels in T cells following CD3/CD28 activation compared with naive T and Tex cells (Figure 2A), which is consistent with previous reports suggesting an elevation of 2-HG levels upon TCR activation (24) (Figure 2A). To explore whether this change is consistent across both isomeric forms of 2-HG, L (*S*) and D (*R*), we conducted further analysis; only L-2-HG increased upon CD3/CD28 activation, whereas D-2-HG levels were equivalent across all 3 groups (Figure 2B). These relative differences are also shown in Supplemental Figure 3B, where the relative content of 2-HG was assessed by comparing the area under the curve of the 3 groups. Similarly, when we sorted PD1^+^ and PD1^–^ CD8^+^ T cells from peripheral blood of patients with HIV and leukemia using flow cytometry, we observed a decreasing trend in 2-HG levels, particularly a decrease in L-2-HG, which aligns with our in vitro experimental results (Figure 2C).

**Figure 3:**
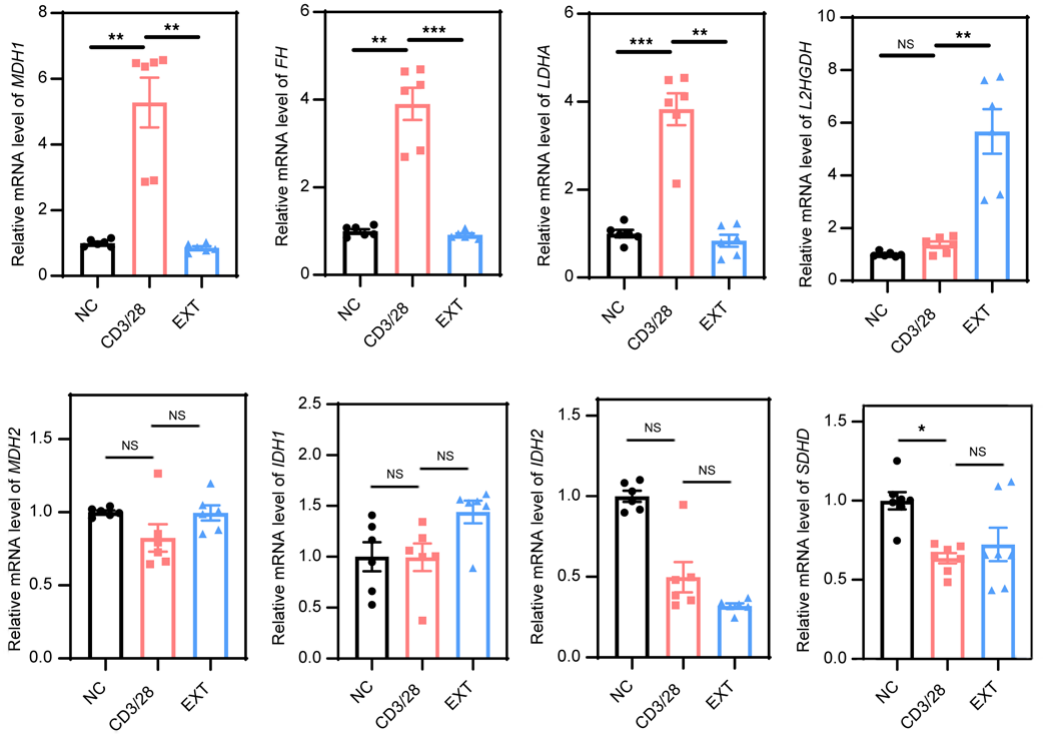
The expression of metabolic genes associated with the Krebs cycle in Tex cells. Detection of the mRNA levels of *MDH1*, *MDH2*, *FH*, *LDHA*, *IDH1*, *IDH2*, *SDHD*, and *L2HGDH* genes in NC, CD3/CD28-activated, and exhaustion group. *n* ≥ 6 per group. Each point represents 1 sample. Data are represented as mean ± SEM. *P < 0.05; **P < 0.01; ***P < 0.001 by 1-way ANOVA. MDH1, malate dehydrogenase 1; MDH2, malate dehydrogenase 2; FH, fumarate hydratase; IDH1, isocitrate dehydrogenase [NADP(+)] 1; IDH2, isocitrate dehydrogenase [NADP(+)] 2; LDHA, lactate dehydrogenase A; SDHD, succinate dehydrogenase complex subunit D; L2HGDH, L-2-hydroxyglutarate dehydrogenase.

To gain insights into how T cells dynamically regulate the concentration of the metabolite L-2-HG, we examined metabolic enzymes associated with the Krebs cycle (Figure 3). We found that the mRNA levels of certain key enzymes involved in the TCA cycle, including *MDH1*, *FH*, and *LDHA*, were upregulated during T cell activation but downregulated in Tex cells (Figure 3). The expression levels of *MDH2*, *IDH1*, *IDH2*, and *SDHD* remained unchanged (Figure 3). Notably, the metabolic enzyme L-2-hydroxyglutarate dehydrogenase (L2HGDH), responsible for degrading the L-2-HG metabolite, showed an upregulated mRNA level specifically in Tex cells (30). Thus, the observed changes in the transcriptional levels of these enzymes contribute to the elevated L-2-HG level in Teff cells but a decreased L-2-HG level in Tex cells.

Previous studies have highlighted the impact of tumor metabolites on the process of T cell exhaustion. It is worth noting that recurrent mutations in IDH have been identified in various tumors (31). The mutated form of IDH promotes the conversion of α-KG to the cancer-associated metabolite R-2-HG (i.e., D-2-HG). It has been demonstrated that R-2-HG can inhibit T cell activity and function (22). On the other hand, L-2-HG has been shown to indirectly impact the expression of CD62L in T cells by regulating DNA methylation. This, in turn, affects T cell activation and differentiation processes (24). However, the specific role of 2-HG in Tex cells has not been extensively studied.

### L-2-HG inhibits TOX expression by changing H3K27me3 modification and chromatin accessibility.

Tex cells represent a unique cellular state characterized by specific epigenetic and transcriptional features. Emerging evidence suggests that these cells possess a distinct epigenetic landscape that plays a role in their diminished responsiveness to immunotherapies (11, 32, 33).

**Figure 4:**
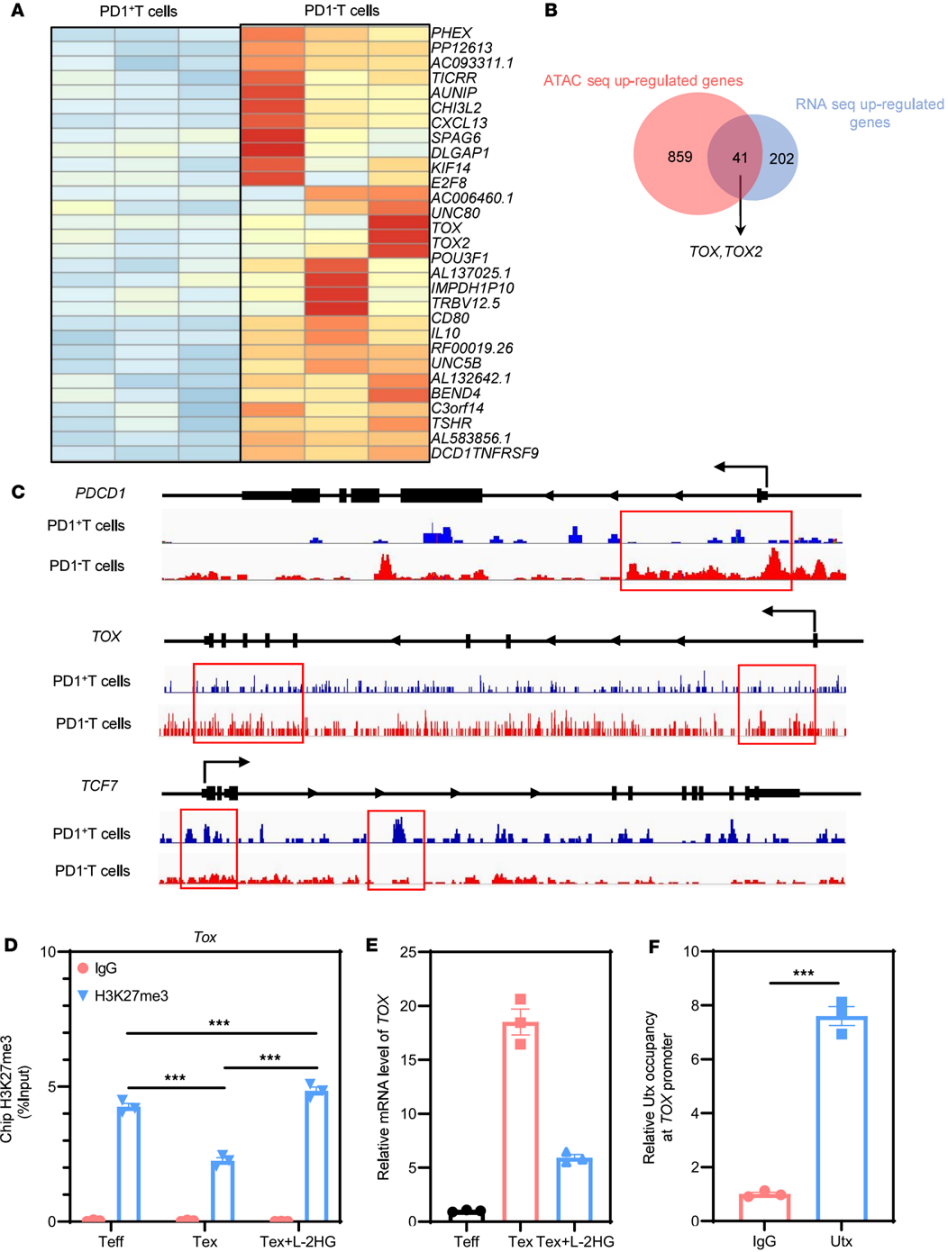
Epigenetic reprogramming of Tex cells. (**A**) Gene expression heatmap of PD1^–^ and PD1^+^ T cells isolated from PBMCs of patients with HIV. (**B**) Venn diagrams depicting overlap between the upregulated genes (*n* = 202) and open chromatin accessibility (*n* = 859) in PD1^+^ T cells. (**C**) Genome browser tracks of ATAC-seq data at the *PDCD1*, *TOX*, and *TCF7* loci in PD1^–^ and PD1^+^ T cells. (**D**) L-2-HG treatment could rescue the H3K27me3 modification at the *TOX* promoter in Tex cells and Teff cells, as determined by ChIP-qPCR. IgGs were included as negative controls for ChIP-qPCR. (**E**) L-2-HG treatment inhibits the mRNA expression of *TOX* in Tex cells. (**F**) Relative UTX occupancy at the *TOX* promoter in Tex cells, as determined by UTX ChIP-qPCR. IgGs were included as negative controls. *n* = 3 per group. Data are represented as mean ± SEM. ***P < 0.001 by 1-way ANOVA. *PDCD1*, programmed cell death 1; *TOX*, thymocyte selection–associated high-mobility group box; *TCF7*, T cell factor 1; UTX, ubiquitously transcribed tetratricopeptide repeat, X chromosome (also known as lysine demethylase 6A [KMD6A]).

To gain insights into the epigenetic and transcriptional changes in Tex cells resulting from chronic HIV infection, we conducted RNA sequencing (RNA-seq) and ATAC sequencing (ATAC-seq) on PD1^+^ and PD1^–^ CD8^+^ T cells isolated from the peripheral blood mononuclear cells (PBMCs) of patients with HIV. Our RNA-seq analysis revealed significant changes in the expression of 871 genes in PD1^+^CD8^+^ T cells compared with PD1^–^CD8^+^ T cells (*P* < 0.05; Supplemental Table 2). Among these genes, 243 were upregulated, including important co-suppressor molecules such as *PDCD1*, *TOX*, and *TOX2* (Figure 4A). In addition, ATAC-seq analysis identified significant changes in chromatin accessibility in PD1^+^CD8^+^ T cells compared with PD1^–^CD8^+^ T cells, with hundreds of genes showing changes (*P* < 0.05; Supplemental Tables 3 and 4). Among the upregulated genes, 41 exhibited altered chromatin accessibility, with *PDCD1* showing the most significant difference, followed by *IL10*, *TOX*, *ICOS*, and others (Figure 4, B and C). We specifically highlighted the comparisons of *PDCD1*, *TOX*, and *TCF7* (which encodes T cell factor 1 protein, TCF1) in terms of chromatin accessibility between PD1^+^ and PD1^–^ CD8^+^ T cells (Figure 4C). The transcription factors TOX and TCF1 have emerged as key drivers of exhaustion and stemness programs in CD8^+^ T cells. These findings provide a further understanding of the epigenetic and transcriptional landscape of Tex cells in chronic HIV infection, shedding light on the underlying mechanisms of T cell exhaustion and potential therapeutic targets (34). In line with this, we found that *TOX* and *TCF7* exhibited opposite changes in their epigenetic levels in Tex cells from patients with HIV in our ATAC-seq data (Figure 4C).

Histone methylation modifications have been recognized as crucial regulators of T cell differentiation and function, with H3K27me3 associated with gene silencing and chromatin accessibility. We examined histone methylation levels to further investigate the epigenetic landscape of Tex cells.

In our analysis of the exhaustion model, we observed a downregulation of H3K27me3 levels in the promoter region of *TOX*, which coincided with the upregulated expression of *TOX* in Tex cells (Figure 4D). This observation suggests that the decreased H3K27me3 levels may contribute to the enhanced expression of *TOX* in Tex cells. Interestingly, L-2-HG, a small molecule metabolite, has been shown to competitively inhibit α-KG–dependent methylases, which are enzymes responsible for histone methylation. Consequently, L-2-HG emerged as a significant regulator of histone methylases in the context of epigenetics. Its ability to influence epigenetic changes provides a potential mechanistic link between metabolic changes and the dysregulated histone methylation observed in Tex cells. These findings highlight the intricate interplay between metabolism, epigenetics, and T cell exhaustion, emphasizing the importance of studying the metabolic and epigenetic regulation of Tex cells for a comprehensive understanding of their functional impairments and potential therapeutic interventions (19). To investigate this further, we treated Tex cells with L-2-HG and observed that it rescued the H3K27me3 levels in the *TOX* promoter and inhibited the expression of *TOX* in Tex cells (Figure 4E). Ubiquitously transcribed tetratricopeptide repeat, X chromosome (UTX; also known as lysine demethylase 6A [KMD6A]) and the Jumonji domain–containing protein 3 (JMJD3) are histone demethylases that regulate the trimethylation of histone H3 on lysine 27 (H3K27me3). We acquired T cell–related ChIP-seq and ATAC-seq datasets from the NCBI Gene Expression Omnibus (GEO) database (accession numbers GSE70795, GSE89036, GSE161842, and GSE64832). The analysis indicates that H3K27Me3 ChIP-seq enrichment at the *Tox* locus was increased in *Utx*- and *Jmjd3*-knockout T cells, suggesting that *Utx* and *Jmjd3* may bind to the *Tox* locus (Supplemental Figure 4A). Methylation modifications at the *Tox* locus are associated with significant changes in T cell differentiation. However, minimal changes in chromatin accessibility in *Jmjd3*-knockout T cells suggest that H3K27me3 modification at the *Tox* locus might not be regulated by *Jmjd3* (Supplemental Figure 4B). Subsequent analysis of the human *UTX* ChIP-seq dataset revealed that *UTX* binds to the *TOX* locus (Supplemental Figure 4C). Next, in Figure 4F, we performed ChIP-PCR experiments to validate the binding to the *UTX* and *TOX* promoters. The data showed that in comparison with IgG control, *UTX* occupancy at the *TOX* promoter in Tex cells was increased.

### L-2-HG improves T cell effector function and mitochondrial metabolism.

**Figure 5:**
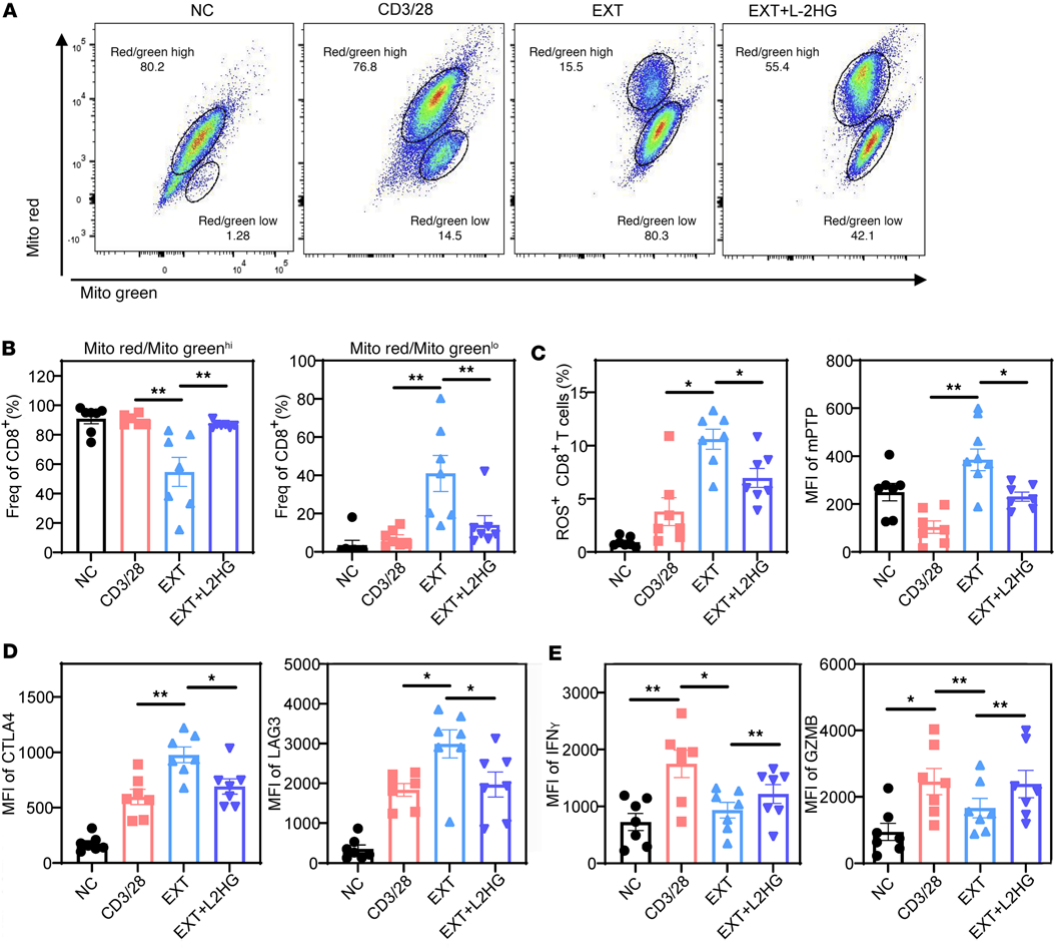
L-2-HG improves T cell effector function and mitochondrial metabolism. (**A** and **B**) Representative flow cytometry of MitoGreen and MitoRed to detect mitochondrial mass and membrane potential in model exhausted T cells treated with L-2-HG (300 μM). (**C**) L-2-HG treatment rescued the high level of ROS and open mPTP in Tex cells. (**D**) L-2-HG treatment inhibited the protein level of co-suppressive molecules CTLA4 and LAG3 in Tex cells. (**E**) L-2-HG treatment rescued the IFN-γ and GZMB level in Tex cells. *n* = 7 per group and each point represents 1 sample. Data are represented as mean ± SEM. *P < 0.05; **P < 0.01 by 1-way ANOVA.

In our study, we utilized an in vitro model to investigate the effects of L-2-HG on cell function and mitochondrial polarization in Tex cells. Flow staining analysis revealed compelling results, demonstrating the impact of L-2-HG treatment on various aspects of Tex cell biology. First, we observed a significant increase in the population of MR/MG^hi^ cells, indicating improved mitochondrial polarization, in Tex cells after L-2-HG treatment (Figure 5, A and B). L-2-HG treatment did not affect apoptosis in the cells (Supplemental Figure 5A). Meanwhile, we tested the effects of L-2-HG with different doses on cell mitochondrial function and *PDCD1* expression. The data suggested that L-2-HG treatment increased the MitoRed/MitoGreen ratio and decreased *PDCD1* expression in a dose-dependent manner (Supplemental Figure 5B). Furthermore, L-2-HG treatment led to a notable reduction in the proportion of ROS^+^ CD8^+^ cells and the opening of mPTP in Tex cells (Figure 5C). These results suggest that L-2-HG treatment can mitigate oxidative stress and maintain mitochondrial integrity in Tex cells. In terms of the expression of co-suppressive molecules, we found that L-2-HG treatment only slightly altered the expression of CTLA4, but not LAG3, in Tex cells (Figure 5D). This suggests that the effects of L-2-HG on co-suppressive molecules may be specific to certain molecules and not universally applicable.

Importantly, L-2-HG treatment resulted in improved expression of the effector molecules IFN-γ and GZMB in Tex cells (Figure 5E). This indicates that L-2-HG has the potential to enhance the effector function of Tex cells, which is a desirable outcome for the restoration of immune responses. Taken together, these findings highlight the potential of L-2-HG as a therapeutic intervention to modulate mitochondrial polarization, mitigate oxidative stress, and enhance effector function in Tex cells. Further exploration of the underlying mechanisms and in vivo studies are warranted to validate the clinical relevance and therapeutic potential of L-2-HG in the context of T cell exhaustion.

### L-2-HG treatment promotes antitumor immunity in mice.

**Figure 6:**
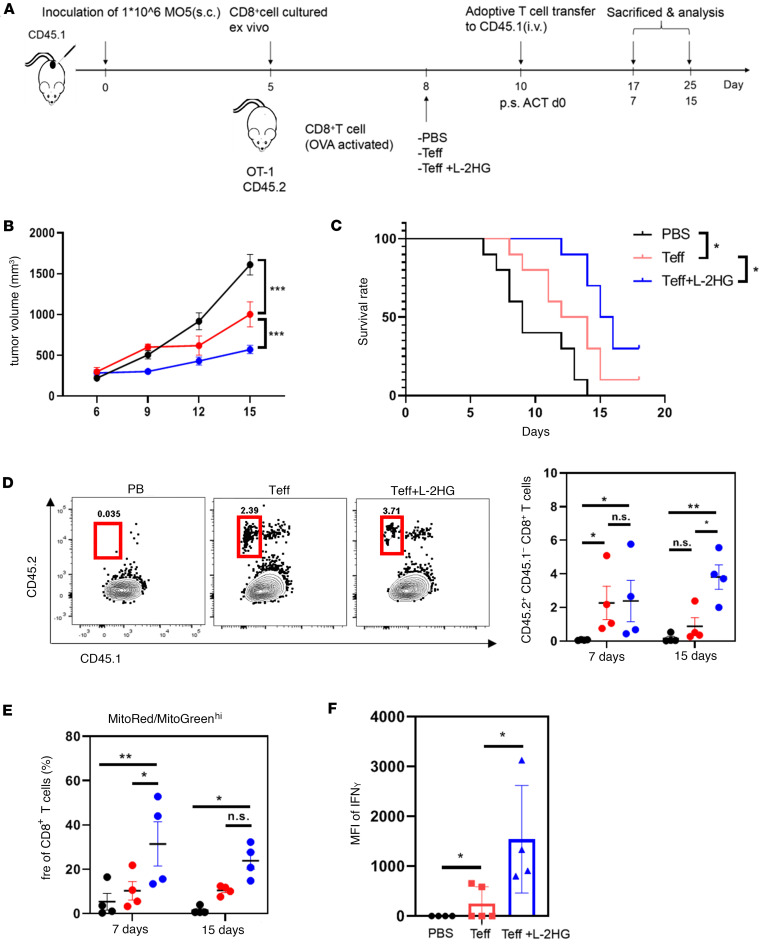
L-2-HG treatment promotes antitumor immunity of TILs. (**A**) Schematic diagram of adoptive transfer of PBS, Teff cells, or Teff cells plus L-2-HG into MO5 melanoma tumor–bearing CD45.1 mice on day 10 after inoculation. (**B** and **C**) CD45.1 mice were subcutaneously injected with MO5 melanoma cells and adopted with CD45.2 CD8^+^ T cells treated with PBS or L-2-HG. Tumor growth curves and survival of the mice were measured at indicated time points. (**D**) At 7 or 15 days after transfer, the number of transferred cells (CD45.1^–^CD45.2^+^ T cells) in the tumors of recipient mice were assessed using flow cytometry. PB, peripheral blood. (**E**) The mitochondrial status of tumor-infiltrating T cells labeled with MitoRed and MitoGreen at 7 days and 15 days. (**F**) The expression of IFN-γ in tumor tumor-infiltrating T cells by flow staining. *n* = 4–8 per group and each dot represents 1 sample. Data are represented as mean ± SEM. *P < 0.05; **P < 0.01; ***P < 0.001 by 1-way ANOVA. OVA, ovalbumin aa 257–264 peptide; PBS, PBS negative control; Teff, effector T cells; Teff+L-2-HG, effector T cells treated with 300 μM L-2-HG.

To investigate the in vivo antitumor effects of L-2-HG, we utilized a CD45.1 mouse model where MO5 cells were subcutaneously injected to induce tumor growth. T cells with or without L-2-HG treatment were administered to the mice (Figure 6A). Our results demonstrated that the L-2-HG–treated group exhibited a significant reduction in tumor volume and an extended survival rate compared with the control group (Figure 6, B and C). Furthermore, analysis of tumor-infiltrating T cells revealed a higher proportion of donor-derived T cells (CD45.1^–^CD45.2^+^ T cells) in the L-2-HG treatment group (Figure 6D), indicating enhanced T cell infiltration into the tumor microenvironment.

Building upon our previous experiments (Figure 5, A–C), we conducted further investigations into the mitochondrial status of tumor-infiltrating T cells. We observed an increased proportion of MR/MG^hi^ cells, indicating improved mitochondrial metabolism, in the L-2-HG treatment group (Figure 6E). This suggests that L-2-HG treatment enhanced the mitochondrial function of T cells within the tumor microenvironment. Additionally, we found an augmentation in the secretion of IFN-γ in the L-2-HG–treated group (Figure 6F). Collectively, our data support the notion that L-2-HG treatment enhances the in vivo antitumor activity of T cells. To determine whether L-2-HG treatment could affect cell migration, we performed adoptive cell transfusion experiments and assessed the cell infiltration 28 hours after transfer. In Supplemental Figure 5, C–E, the data revealed that a slight increase in CD45.2^+^ T cell infiltration occurred in spleen and lymph nodes but not in tumor. Meanwhile, the proliferation and effector function of CD45.2^+^ T cells detected by CFSE labeling and IFN-γ staining exhibited no significant change in the 2-HG treatment group.

## Discussion

T cell exhaustion is a complex process involving the interplay between metabolism and epigenetic regulation, and the specific role of this “metabolism-epigenetic” mechanism in T cell exhaustion remains poorly understood. In the current study, we investigated the metabolic profile of Tex cells, in comparison with Teff cells, and found a distinct feature of significantly reduced L-2-HG levels. While the relationship between L-2-HG and T cells is still relatively unexplored, previous research suggests that L-2-HG levels can be modulated by TCR activation and can be regulated through a HIF1α-dependent mechanism in mouse CD8^+^ T cells (24). Additionally, treatment with L-2-HG could enhance the antitumor capacity of CAR-T cells (35).

Mitochondria, as crucial organelles in cellular metabolism and function, undergo dynamic changes during T cell activation, including processes such as mitochondrial fusion and fission. However, in the tumor microenvironment, TILs exhibit mitochondrial abnormalities. These abnormalities encompass damaged mitochondria, fragmented morphology, increased ROS generation, and impaired OXPHOS metabolism. These observations highlight the impact of the tumor microenvironment on mitochondrial dynamics and metabolism in TILs, which can contribute to T cell dysfunction and exhaustion within the tumor milieu. Understanding the intricate relationship between mitochondrial dysfunction and T cell exhaustion in tumors is essential for developing effective immunotherapeutic strategies (27). Recent studies have provided compelling evidence that CD8^+^ TILs, characterized by terminal exhaustion features including diminished effector function, dysregulated epigenetic landscape with altered chromatin accessibility and CpG methylation patterns, and decreased expression of the transcription factor TCF1, gradually accumulate dysfunctional and depolarized mitochondria (34). In our comprehensive investigation, we made significant discoveries regarding the mitochondrial dysfunction observed in CD8^+^ Tex cells. We observed a notable decline in mitochondrial function, including reduced mitochondrial mass and membrane potential, along with the accumulation of mitochondrial ROS (mtROS). Additionally, we identified abnormal opening and closing of the mPTP in Tex cells.

Based on our findings, we propose that the aberrant opening of mPTP in Tex cells contributes to mitochondrial depolarization and the accumulation of mtROS. However, the exact nature of the relationship between the persistent opening of mPTP and the abnormal mitochondrial metabolism in Tex cells remain to be explored. Further investigations are required to elucidate whether the prolonged opening of mPTP acts as a causative factor or a concurrent consequence of the observed mitochondrial metabolic abnormalities.

Our investigation highlighted the association between metabolic changes, epigenetic reprogramming, and histone methylation changes in Tex cells. Notably, our study demonstrated that the administration of L-2-HG effectively restored the impaired effector function of Tex cells. Furthermore, L-2-HG treatment showed promising results in improving mitochondrial metabolism by elevating the H3K27me3 level, a key histone methylation mark associated with gene regulation. Additionally, we observed a downregulation in the mRNA expression of *TOX*, a transcription factor known to promote the terminal differentiation of CD8^+^ Tex cells, which are typically unresponsive to immunotherapy interventions. Current evidence suggests that these methylation changes are not exclusive to *TOX* but impact a network of genes contributing to the exhaustion phenotype. Notably, genes such as *PDCD1* (PD1), *TCF7* (TCF1), and other transcription regulators like *CD28*, *CCR7*, and *LEF1* are subject to methylation modifications during T cell exhaustion. For instance, chronic viral infections have been shown to enforce demethylation at the *PDCD1* locus, driving persistent expression of PD1, a hallmark of Tex cells (36). Similarly, *TCF7* promoter methylation has been linked to rapid exhaustion in CD8^+^ T cells, while the deletion of *DNMT3A* in CAR-T cells can prevent exhaustion by preserving the stemness of immune cells through transcription regulators such as *TCF7* and *LEF1* (37, 38). These findings underscore that methylation changes influence a broader network of genes, not just *TOX*, and highlight the complexity of the regulatory landscape in T cell exhaustion. Incorporating this expanded understanding into therapeutic strategies may enhance efforts to reverse T cell exhaustion and improve immune function in chronic disease contexts. These findings shed light on the potential therapeutic role of L-2-HG in overcoming the functional limitations of Tex cells, particularly in the context of immunotherapy (39). In our in vivo experiments using MO5 melanoma mice, we observed that the administration of L-2-HG to T cells enhanced their antitumor efficacy, indicating the potential of L-2-HG treatment to positively impact the function of adoptive cells. These findings suggest that L-2-HG may hold promise in delaying or reversing the process of T cell exhaustion through its epigenetic regulation of critical transcription factors in Tex cells.

This research not only uncovers the role of L-2-HG in T cell exhaustion but also provides valuable insights into how mitochondrial depolarization triggers metabolic dysregulation and disturbs the metabolism of essential molecules. Moreover, it establishes a foundation for future investigations into the mechanisms underlying the interaction between mitochondrial depolarization and epigenetic reprogramming of T cell function. We anticipate that this study will contribute to the identification of therapeutic targets and strategies for improving immunotherapy approaches.

## Methods

### Sex as a biological variable.

Sex was not considered as a biological variable. We used only male mice to simplify the experimental design and reduce variability. It is unknown whether the findings are relevant for female mice.

### Human specimens.

This study included the enrollment of 12 patients diagnosed with HCC. Prior to tumor resection, none of the patients underwent chemotherapy or radiation therapy. Paired samples of HCC tumors, adjacent normal tissues (ANTs), and peripheral blood were collected from these patients. The ANTs were located at a distance of at least 3 cm from the corresponding tumor tissues. Additionally, peripheral blood samples were collected from HIV-infected patients and leukemia patients at Shenzhen Third People’s Hospital and Ruijin Hospital of Shanghai Jiao Tong University, respectively.

### Mice.

Male CD45.1 mice at 8–10 weeks of age were obtained from Shanghai Nanfang Model Biotechnology Co., Ltd. Animal experiments were conducted at the Fudan Animal Center following the guidelines for animal welfare. To establish the melanoma xenograft model, 1 × 10^6^ MO5 cells (provided by Shanghai Institute of Immunity and Infection, Chinese Academy of Sciences) suspended in PBS were subcutaneously injected into CD45.1 mice. Tumors became palpable, on average, 10 days after inoculation. CD45.2 OT-1 mouse splenocytes were cultured in a medium containing 10% fetal bovine serum, 12.5% HEPES, 5% sodium pyruvate, and 5% nonessential amino acids, along with 100 U/mL IL-2 (PeproTech, 200-02-50). The splenocytes were then stimulated with 0.1 nM OVA_257–264_ peptide (MCE, HY-P1489A) for 3 days. On the third day, OT-1 CD8^+^ T cells were isolated from the splenocytes using the MojoSort Mouse CD8 T Cell Isolation Kit (BioLegend, 480035). The sorted OT-1 CD8^+^ T cells were treated with L-2-HG (300 μM; Toronto Research Chemicals, H942596) for 3 days, while the Teff group was only treated with IL-2 (100 U/mL). On the sixth day, the L-2-HG–treated T cells and Teff cells were collected and injected into CD45.1 mice through the tail vein on the tenth day after tumor inoculation. Tumor size was measured every 2 days following adoptive transfer, and on days 2 (28 hours), 7, and 15, the mice were sacrificed to collect tumor, lymph node, spleen, and peripheral blood tissues for further analysis.

### FACS analysis.

Cells were stained with antibodies against CD3 (BioLegend, clone HIT3a), CD8 (BioLegend, clone SK1), PD1 (BioLegend, clone EH12.2H7), LAG3 (BioLegend, clone C9B7W), TIM3 (BioLegend, clone A18087E), CTLA4 (BioLegend, clone BNI3), perforin (BioLegend, clone dG9), IFN-γ (BD Pharmingen, clone B27), TNF-α (BioLegend, clone MAb11), CD45.1 (BioLegend, clone QA18A43), CD45.2 (BioLegend, clone 104), CD44 (BioLegend, clone 7A6), CD62L (BioLegend, clone QA18A42), and GZMB (BioLegend, clone GB11), followed by incubation with secondary antibodies conjugated to various fluorochromes; or with MitoTracker Red CMXRos (Thermo Fisher Scientific, M7512), MitoTracker Green FM (Thermo Fisher Scientific, M7514), TMRM (Thermo Fisher Scientific, M20036), or MitoSox Red (Thermo Fisher Scientific, M36008). The degree of openness of mPTP was detected according to the instructions of the mPTP kit (Thermo Fisher Scientific, I35103). FACS analysis was conducted on a BD FACSAria II flow cytometer and FlowJo v10 was used for analysis.

### GC-MS analysis of metabolites.

Cell number and cell diameter were quantified using an automated cell counter (Countstar). Immediately after cell harvesting, 1 mL of 80% (v/v) cold (–80°C) methanol was added to fix the cells. Lyophilized samples were subjected to overnight oximation at 37°C with 30 μL of pyridine containing 20 mg/mL methoxylamine hydrochloride (Sigma-Aldrich, 226904), followed by incubation at 70°C for 30 minutes. After filtration, cell extracts were analyzed using a UHPLC system (Acquity UPLC I-Class, Waters) coupled with a triple quadrupole mass spectrometer (Xevo TQ-XS, Waters). The relative concentrations of metabolites were determined based on the peak areas obtained from the UHPLC-MS analysis.

The UHPLC-MS/MS analysis was conducted using an Agilent 1290 Infinity II UHPLC system coupled to a 6470A triple quadrupole mass spectrometer. Samples were injected onto a Syncronis C18 column (100 mm × 2.1 mm, 1.7 μm, Thermo Fisher Scientific) at a flow rate of 0.2 mL/min, with an injection volume of 10 μL. The mobile phase consisted of water with 5 mM ammonium formate, 0.01% formic acid (phase A), and acetonitrile (phase B). A gradient elution program was used for chromatographic separation as follows: 0–3 minutes, 1% B; 6 minutes, 15% B; 7–9 minutes, 99% B; 9.1–11 minutes, 1% B. Electrospray ionization in negative mode (ESI^–^) was employed for ionization of the eluted analytes. The source drying gas and sheath gas were maintained at temperatures of 300°C and 350°C, respectively. The flow rates of the source drying gas and sheath gas were set at 5 and 11 L/min, respectively. The nebulizer pressure was 35 psi, and the capillary voltage was 3000 V. MS detection was carried out using multiple reaction monitoring (MRM) with optimized fragment and collision energy under negative ion mode. The MRM transitions (precursor ions → product ions) used were 363→147 and 363→129 for DATAN-labeled 2-HG, and 368→152 and 368→134 for the DATAN-labeled internal standard. The retention times for DATAN-labeled L-2-HG (or L-2-HG-^13^C_5_) and D-2-HG (or D-2-HG-^13^C_5_) were 4.14 minutes and 4.88 minutes, respectively. Data acquisition and instrument control were performed using MassHunter software (version B.08.00, Agilent). Data were integrated and the output peak area of each target substance was used to quantify metabolite labels based a standard curve generated from an internal standard for the on-machine test sample, and further normalized to the content of the cell sample.

### RT-qPCR.

Total RNA was extracted from T cells using TRIzol reagent (Thermo Fisher Scientific) and reverse transcribed according to the manufacturer’s instructions. Real-time PCR was performed using gene-specific primers and SYBR Premix Ex Taq (TaKaRa) as per the manufacturer’s protocol. *ACTB* (β-actin) was used as the housekeeping control gene. The primer sequences can be found in Supplemental Table 1.

For RNA-seq analysis, PD1^–^CD3^+^CD8^+^ and PD1^+^CD3^+^CD8^+^ cells were sorted from peripheral blood of patients with HIV using flow cytometry (BD FACSAria II). After counting and centrifugation, the cells were frozen in TRIzol. High-throughput mRNA-seq and analysis were performed by Novogene Technology Co., Ltd. The quality of T cell total RNA was assessed using the BioAnalyzer 2100 (Agilent Technologies). RNA libraries were constructed using the NEBNext Ultra RNA Library Prep Kit (New England Biolabs), and library size was evaluated for quality control. RNA quantification was conducted using a Qubit fluorometer (Thermo Fisher Scientific). The libraries were sequenced on an Illumina HiSeq sequencer following the manufacturer’s instructions.

For ATAC-seq analysis, PD1^–^CD3^+^CD8^+^ and PD1^+^CD3^+^CD8^+^ cells from the peripheral blood of patients with HIV were sorted using flow cytometry (BD FACSAria II). The ATAC-seq kit was obtained from Nanjing Wanzhi Biotechnology Company. A total of 5 × 10^4^ cells from each sample were collected, centrifuged, and placed on ice. Then, 50 μL of the lysate (10 mM Tris·HCl pH 7.4, 10 mM NaCl, 3 mM MgCl_2_, 0.1% [v/v] IGEPAL CA-630 [Sigma-Aldrich, 18896]) was added, followed by centrifugation at the same speed to collect the nuclei. ATAC-seq libraries were constructed using the Norway Zan kit and sequenced using 75-bp paired-end reads after size selection on an Illumina HiSeq 2000.

Reads were trimmed and aligned to the reference human genome (UCSC hg38) using Bowtie2 (version 2.5.1; https://bowtie-bio.sourceforge.net/bowtie2/index.shtml). Quality filtering and removal of duplicates were performed, and reads were mapped to the M chromosome using SAMtools (https://www.htslib.org) and Picard tools (https://broadinstitute.github.io/picard). For downstream analysis, read counts were normalized to 1× depth (reads per genome covered, RPGC) using the “bamCoverage” function of deepTools28 (http://deeptools.ie-freiburg.mpg.de). Peak calling was conducted using the “call peak” function of MACS2 (version 2.2.7.1; http://liulab.dfci.harvard.edu/MACS) with the following parameters: --nomodel --shift -100 --extsize 200 -q 0.05. Signaling trajectories were visualized using the UCSC Genome Browser (https://genome.ucsc.edu/).

### ChIP-qPCR.

The ChIP-qPCR assay was conducted using the SimpleChIP Enzymatic Chromatin IP kit (Cell Signaling Technology, 9002) following the manufacturer’s protocol. CD8^+^ Tex cells were crosslinked with 1% paraformaldehyde for 10 minutes at room temperature, and the crosslinking reaction was quenched by adding 2.5 M glycine to a final concentration of 0.125 M for 5 minutes. The cells were lysed, and chromatin fragments of 150 to 700 bp were obtained by digestion with micrococcal nuclease. The solubilized chromatin was incubated with antibodies against UTX (Cell Signaling Technology, catalog 33510) or control IgG at 4°C for 3 hours, followed by incubation with Protein A beads. The antibody-chromatin complexes were pulled down and washed with CHIP buffer. After de-crosslinking by proteinase, the immunoprecipitated DNA was extracted using a PCR Purification Kit (QIAGEN). The DNA fragments were analyzed by qPCR using specific primers for the *TOX* promoter, as listed in Supplemental Table 1.

### Primary human T cell isolation and in vitro activation.

For primary human T cell isolation and in vitro activation, PBMCs from healthy donors were isolated using density gradient centrifugation (Ficoll, GE Healthcare). T cell subsets were isolated from PBMCs using appropriate magnetic beads (BioLegend) according to the manufacturer’s protocol. The isolated T cells were cultured in X-VIVO 15 culture medium (Lonza). Human T cells were activated with anti-CD3 (clone UCHT1, 2 μg/mL, BioLegend) and anti-CD28 (clone CD28.2, 2 μg/mL, BioLegend).

### In vitro CD8^+^ Tex cells.

To generate in vitro CD8^+^ Tex cells, purified human CD8^+^ T cells were stimulated with anti-CD3 (2 μg/mL) for 3–4 days, followed by additional restimulations every 3–4 days using anti-CD3 (1 μg/mL) and anti-CD28 (2 μg/mL) to generate CD8^+^ Tex cells. CD8^+^ Tex cells were then treated with or without 2-HG.

### ChIP-seq and ATAC-seq data processing.

ChIP-seq and ATAC-seq datasets were acquired from the GEO database (accession numbers GSE70795, GSE89036, GSE161842, and GSE64832). The adaptor sequences were removed using Cutadapt (version 3.5; http://pypi.python.org/pypi/cutadapt) to ensure data quality. Cleaned reads were then mapped to the human (hg19) and mouse (mm10) reference genomes using Bowtie2. Peak calling for the mapped data was performed using MACS2 with default parameters. Peak regions were converted from bedgraph to bigwig format using bedClip (http://hgdownload.soe.ucsc.edu/admin/exe/linux.x86_64), bedtools (http://code.google.com/p/bedtools), and bedGraphToBigWig (http://hgdownload.soe.ucsc.edu/admin/exe/linux.x86_64) for visualization by the Integrative Genomics Viewer (IGV; https://igv.org).

### Statistics.

Statistical analyses were performed using GraphPad Prism 8 software. For comparisons between 2 independent groups, an unpaired, 2-tailed Student’s *t* test was performed, while paired data were assessed using a paired, 2-tailed Student’s *t* test. Multiple comparisons were assessed with 1-way ANOVA, and grouped data were assessed by 2-way ANOVA. The statistical significance level was defined as a *P* values of less than 0.05. Error bars show SEM.

### Study approval.

The study received approval from the ethics committee at Zhongshan Hospital, Fudan University, and written informed consent was obtained from patients.

### Data availability.

The datasets used or analyzed during the current study are available from the corresponding author upon reasonable request. The raw sequence data reported in this paper have been deposited in the Genome Sequence Archive in National Genomics Data Center, China National Center for Bioinformation/Beijing Institute of Genomics, Chinese Academy of Sciences (GSA-Human: HRA006727) that is publicly accessible at https://ngdc.cncb.ac.cn/gsa-human (40). Raw data for all graphs are reported in the Supporting Data Values file.

## Author contributions

DW contributed to the conception of the study, project administration, and funding acquisition. Yanying Yang, Xiaoyan Li, FL, MM, ZC, and Ying Yang performed experiments, analyzed data, and wrote the manuscript. XW, CR, and YL contributed to the construction of the sequencing database. HW contributed to the conception of the study and project administration. Xiaoyang Li, YS, and ZZ helped collect clinical samples and perform the analysis with constructive discussion.

## Supplementary Material

Supplemental data

Supplemental table 1

Supplemental table 2

Supplemental table 3

Supplemental table 4

Supporting data values

## Version 1 03/05/2025

In-Press Preview

## Version 2 04/08/2025

Electronic publication
