# Supplementary material for: Immunometabolite L-2-HG promotes epigenetic modification of exhausted T cells and improves antitumor immunity
Source: JCI Insight. 2025 Mar 4;10(7):e174600. doi: 10.1172/jci.insight.174600 (PMC11981629; doi:10.1172/jci.insight.174600)
Supplement: Supplemental data [file jciinsight-10-174600-s102.pdf]

Supplemental Figure. 1

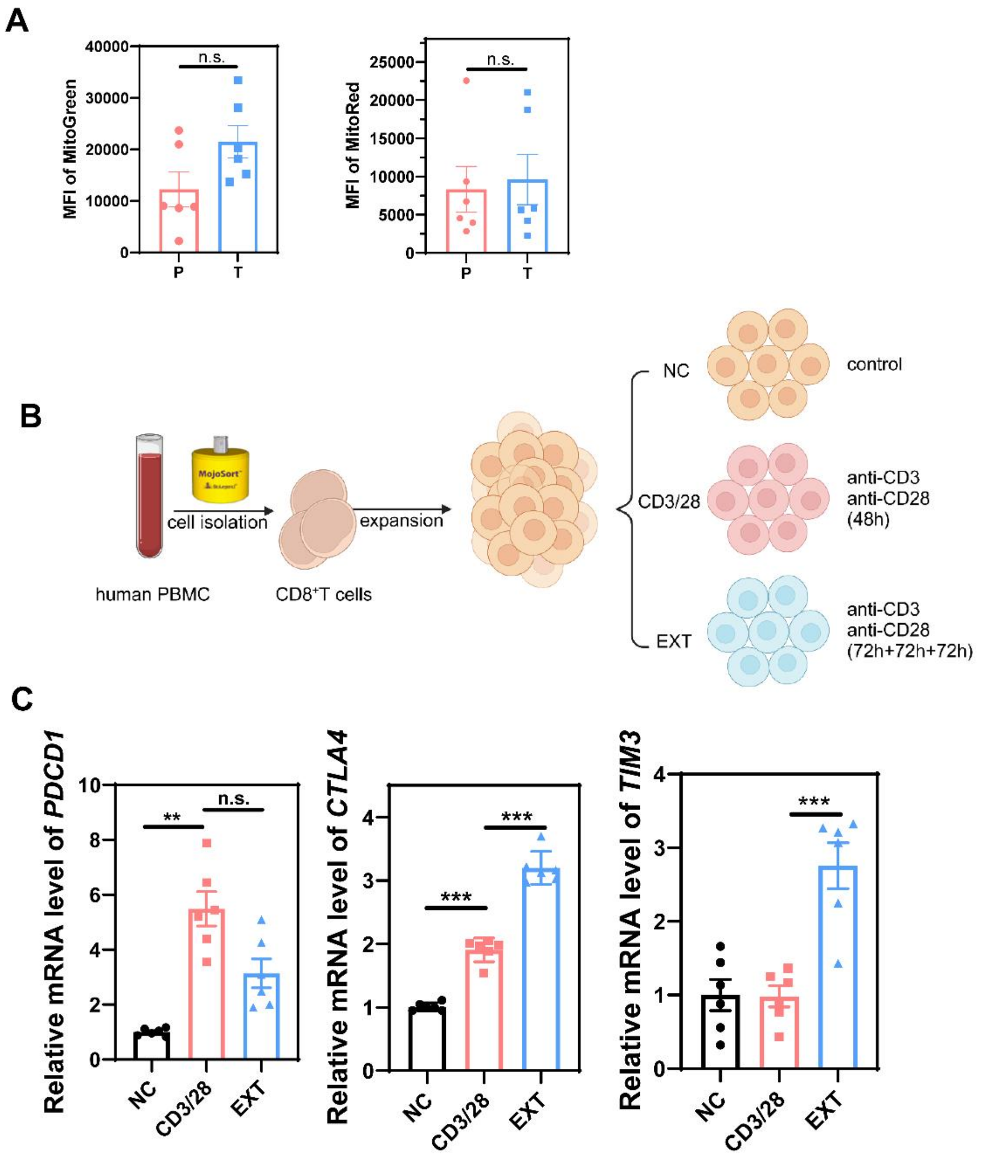

Supplemental Figure 1. Exhaustion model construction and evaluation

A. Bar graph showing MFI of MitoGreen and MitoRed in T cells of tumor tissue and paracancerous tissue of HCC patients.

B.Scheme of the in vitro exhaustion model construction.

C.The mRNA expression levels of *PDCD1, CTLA4, TIM3* genes in NC, CD3/28 activation, and exhaustion.

n=6 for per group and each dot represents a sample(A,C). Data are represented as mean ± SEM. * $P < 0.05$, ** $P < 0.01$, *** $P < 0.001$ by 1-way ANOVA (C) or 2-tailed Student’s t test (A). PDCD1, Programmed cell death protein 1; CTLA4, Cytotoxic T-Lymphocyte Associated Protein 4; TIM3, hepatitis A virus cellular receptor 2.

Supplemental Figure. 2

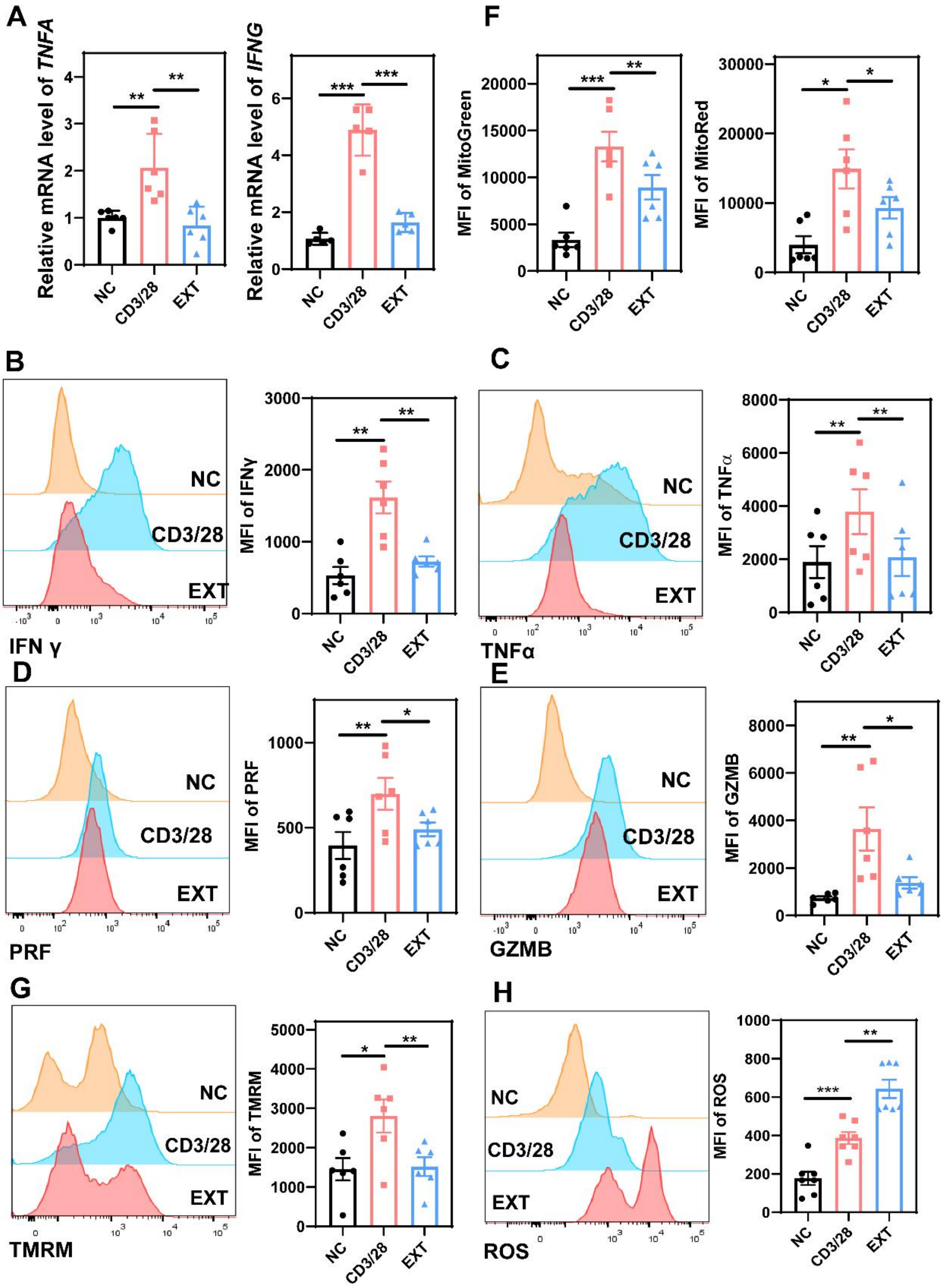

Supplemental Figure 2. Exhaustion model phenotype

A.The mRNA expression levels of *TNFA, IFNG* in NC, CD3/28 activation, and exhaustion group.

B-E.The protein level of IFNγ, TNFα, PRF1, and GZMB in NC, CD3/28 activation, and exhaustion group.

F. Histogram showing MFI stained with MitoGreen and MitoRed in cells of NC, CD3/28 activation, and exhaustion group. G and H. The representative flow chart of TMEM (left) and ROS (right) levels of T cells in the NC group, CD3/28 activation group, and exhaustion group. n=4-7 for per group and each dot represents a sample(A-G). Data are represented as mean ± SEM. * $P < 0.05$, ** $P < 0.01$, *** $P < 0.001$ by 1-way ANOVA (A-G). IFNγ, interferon gamma; TNFα, tumor necrosis factor alpha-like; PRF1, perforin 1; GZMB, granzyme B; TMRM, tetramethylrhodamine, methyl ester; ROS, Reactive oxygen species.

Supplemental Figure. 3

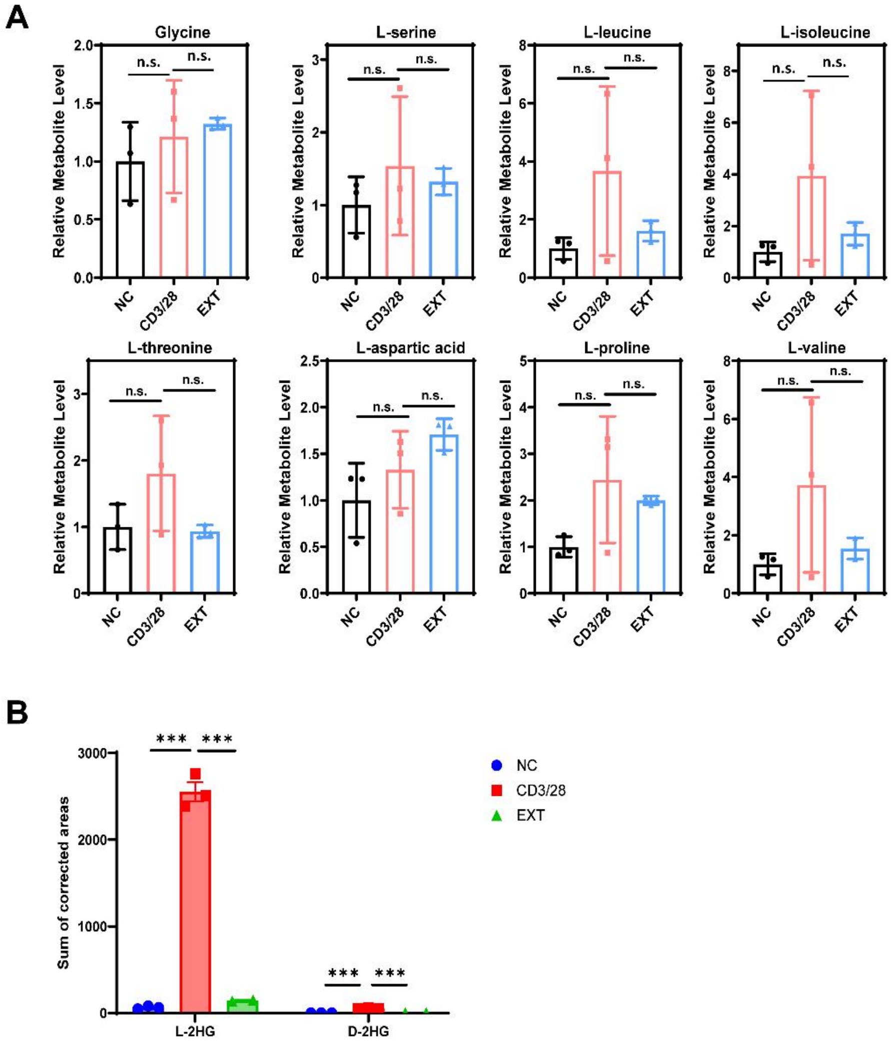

Supplemental Figure 3. Exhausted T cell metabolic imbalance

A. Detection of amino acid in NC, CD3/28 activation, and exhaustion group by GC-MS. N=3 per group. Detection of the relative content of 2-HG levels by GC-MS through the area under the curve in NC, CD3/28 activation, and exhaustion group. Data are represented as mean ± SEM. * $P < 0.05$, ** $P < 0.01$, *** $P < 0.001$ by one-way ANOVA.

Supplemental Figure 4

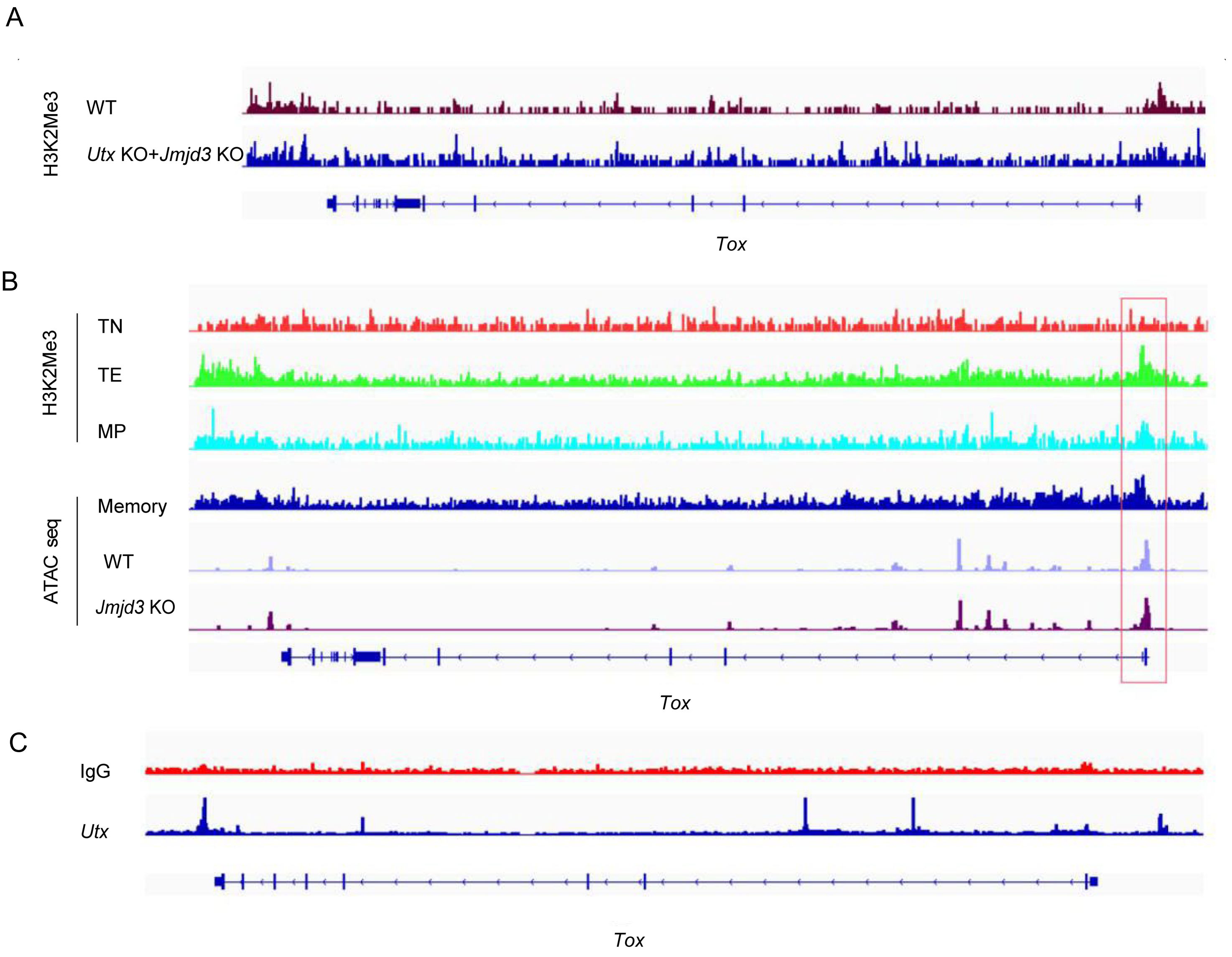

A. ChIP-seq analysis reveals an increase in H3K27Me3 modification at the *Tox* locus following *Utx* and *Jmjd3* knockout in OT-II Kdm6b$^{f/f}$, Kdm6a$^{f/f}$ Cd4^{-}Cre mature CD4 SP thymocytes.

B. Differential H3K27Me3 modification levels at the *Tox* locus across various CD8^{+} T cell subsets, including naive, KLRG1^{hi}IL7R^{lo} terminal effector, KLRG1^{lo}IL7R^{hi} memory precursor, and KLRG1^{lo}IL7R^{hi} memory CD8^{+} T cells, are shown. ATAC-seq analysis of chromatin accessibility in WT and *Jmjd3* knockout CD8^{+} T cells (P14 TCR transgenic) is also presented.

C. ChIP-seq analysis demonstrates UTX binding at the *TOX* locus in the T-ALL cell line Jurkat.

The datasets were obtained from the GEO database (accession numbers GSE70795, GSE89036, GSE161842, and GSE64832 respectively)

Supplemental Figure.5

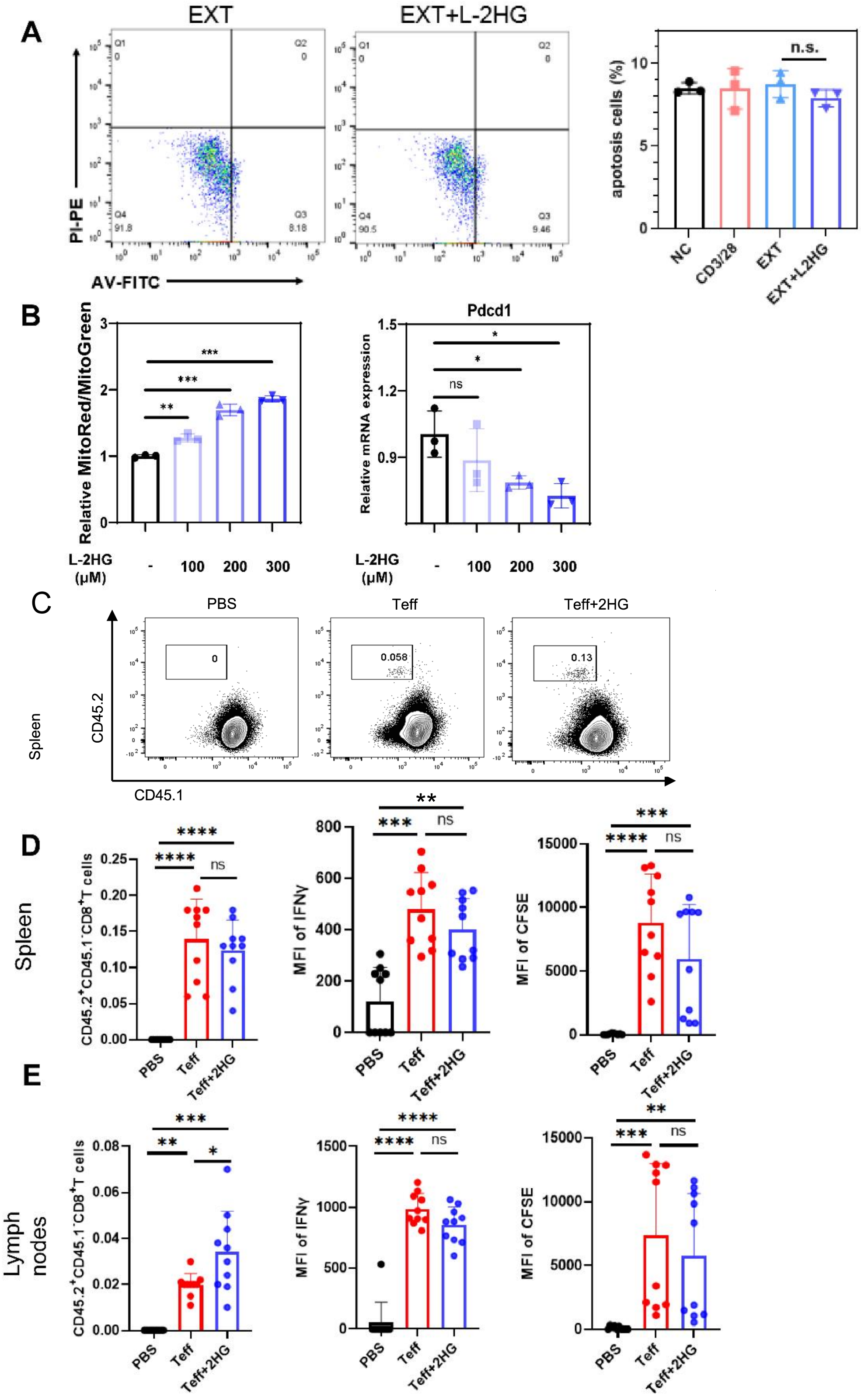

Supplemental Figure 5. The effects of L-2HG treatment on exhausted T cells phenotypes and anti-tumor immunity

A. Apoptosis assessment by Annexin V/ Propidine Iodide (PI) staining of exhausted model T cells treated with +/- L-2HG (n=3 for each group). The methods have been reported in Figure 5.

B. We tested the effects of L-2HG with different doses on cell mitochondrial function and *PDCD1* expression (n=3 for each group).
C-E. At 28 hours post transfer, the number of transferred cells ($CD45.2^+CD45.1^-$ T cells), the expression of IFN-γ and CFSE staining in the spleens(C-D) or lymph nodes(E) of recipient mice were assessed using flow cytometry. The detailed methods have been illustrated in Figure 6. n=10 for each group(D-E).
Data are represented as mean ± SEM. * $P < 0.05$, ** $P < 0.01$, *** $P < 0.001$ by 1-way ANOVA. PBS, PBS negative control; Teff, effector T cells; Teff+L-2HG, effector T cells treated with 300μM L-2HG.

Supplemental Table 1. The primer sequences of genes tested in the study.
Supplemental Table 2. The list of 871 genes with significant expression changes tested by RNA sequencing analysis ($p < 0.05$) in $PD1^+$ $CD8^+$ T cells compared to $PD1^-$ $CD8^+$ T cells.
Supplemental Table 3 and 4. ATAC sequencing analysis identified significant alterations in chromatin accessibility in $PD1^+$ $CD8^+$ T cells compared to $PD1^-$ $CD8^+$ T cells, with hundreds of genes showing changes ($p < 0.05$); Supplemental Table 3 for up-regulated peaks and Supplemental Table 4 for down-regulated peaks.
